# Modulation and Evolution of Animal Development through microRNA Regulation of Gene Expression

**DOI:** 10.3390/genes10040321

**Published:** 2019-04-25

**Authors:** Sebastian Kittelmann, Alistair P. McGregor

**Affiliations:** 1 Sir William Dunn School of Pathology, University of Oxford, South Parks Road, Oxford, OX1 3RE, UK; sebastian.kittelmann@path.ox.ac.uk; 2 Department of Biological and Medical Sciences, Oxford Brookes University, Gipsy Lane, Oxford, OX3 0BP, UK

**Keywords:** microRNA, development, evolution, gene regulation

## Abstract

microRNAs regulate gene expression by blocking the translation of mRNAs and/or promoting their degradation. They, therefore, play important roles in gene regulatory networks (GRNs) by modulating the expression levels of specific genes and can tune GRN outputs more broadly as part of feedback loops. These roles for microRNAs provide developmental buffering on one hand but can facilitate evolution of development on the other. Here we review how microRNAs can modulate GRNs during animal development as part of feedback loops and through their individual or combinatorial targeting of multiple different genes in the same network. We then explore how changes in the expression of microRNAs and consequently targets can facilitate changes in GRNs that alter development and lead to phenotypic evolution. The reviewed studies exemplify the key roles played by microRNAs in the regulation and evolution of gene expression during developmental processes in animals.

## 1. Introduction

microRNAs are short (19–25 nucleotides) non-coding transcripts that reduce the expression levels of protein-coding genes post-transcriptionally. They act by binding to complementary seed sequences in the mRNAs of target genes in a ribonucleoprotein complex to block translation of the target mRNA and/or promote its degradation (for a recent review see [1]). The hardwiring of microRNAs into GRNs can help to directly regulate particular switches and, consequentially, developmental decisions, and/or to provide more global robustness to the outputs of GRNs in the face of environmental or genetic perturbation [1,2,3]. 

In animals, microRNAs are thought to target the transcripts of thousands of genes and they have even been predicted to target the majority of mRNAs in humans [1,4,5,6]. This means that microRNAs are likely involved in the regulation of most developmental processes in animals [1]. The importance of microRNAs is demonstrated by the fact that the removal of most conserved microRNA families in animals like *Drosophila melanogaster* and *Mus musculus* produces strong phenotypes, often affecting a range of traits, although the loss of others, particularly newer or lineage-specific microRNAs has more subtle or no detectable phenotypes [1,7]. It is clear, therefore, that the fine-tuning of gene expression by microRNAs is not only very important for the regulation of specific individual target genes but also the interactions within and outputs of developmental GRNs more generally. 

It follows that changes in microRNA expression or function can lead to phenotypic evolution [8,9]. The expression, processing and functionality of microRNAs, and the evolution and roles of microRNA families in metazoans have been comprehensively covered in many excellent reviews (e.g., [1,3,8,9,10,11,12]). Here instead we focus on how microRNAs can function in feedback loops and act as switches to target key nodes or multiple components in GRNs to help regulate developmental processes. We also review how changes in microRNAs have facilitated phenotypic evolution and provide a perspective on the roles microRNAs may have played in the evolution of development and the diversification of animals.

## 2. microRNAs in Regulatory Loops

**Figure 1:**
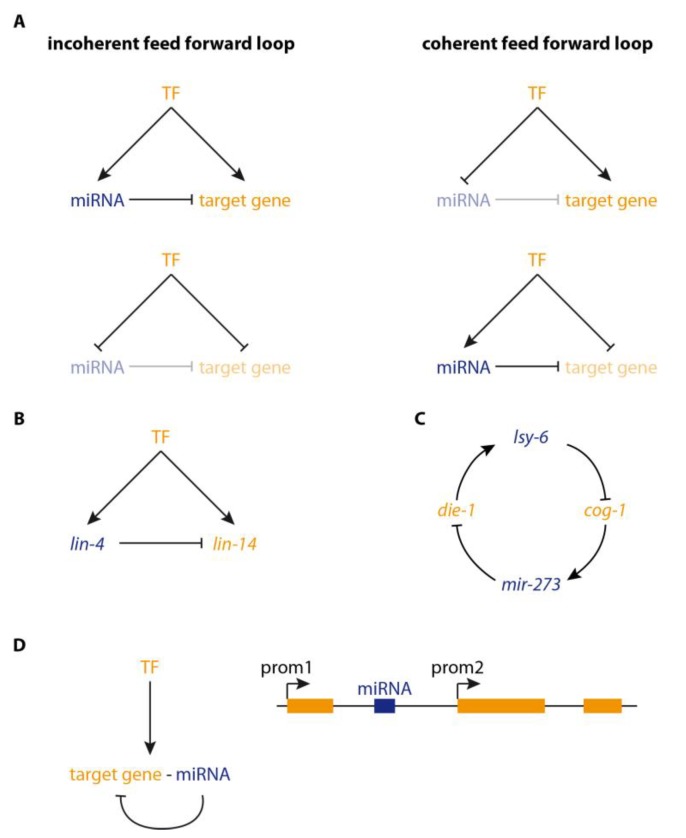
Gene regulatory network (**GRN) sub-circuits involving microRNAs.** Protein-coding genes are coloured orange, microRNAs are blue, with dark and light colouring of the text indicating whether the gene is expressed or not, respectively. (**A**) Feed-forward loops (FFLs) are over-represented within GRNs. In these circuits, expression of a microRNA and its target are regulated by the same upstream transcription factors (TF). In incoherent FFLs, the TF has the same effect on microRNA and target gene resulting in activation or repression of both. In this case, repression by the microRNA leads to the buffering of target expression levels. In coherent FFLs, the TF has opposite effects on microRNA and target gene which leads to mutually exclusive expression. Such FFLs have been implicated in expression switches where, e.g., transcriptional repression of a target gene is reinforced by the activation of a microRNA and, thus, removal of persisting transcripts. (**B**) Repression of *lin-14* by the microRNA *lin-4* is an example for an incoherent FFL. The expression of both genes is temporally coordinated, but no transcriptional activators have to our knowledge been identified. Buffering of the *lin-14* expression level by *lin-4* results in the transition from a cycling expression pattern to a stable temporal expression gradient. For further examples of FFLs see [31]. (**C**) A double negative feedback loop involving microRNAs controls the developmental switch from an equipotent state to the bistable expression of specific genes in the two *C. elegans* taste receptor neurons. (**D**) Intragenic microRNAs are usually co-regulated with their host gene, which is often also a target gene. Expression of the microRNA can be avoided if the host gene has different promoters (prom1, prom2) that can be regulated individually and lead to the expression of different host gene isoforms.

Computational analyses of GRNs have revealed over-represented motifs involving microRNAs [13]. They often act in feed-forward loops (FFL) in which a microRNA and its target gene are regulated by the same transcription factor (TF) [14]. FFLs are categorised into incoherent and coherent FFLs, depending on whether the upstream TF has the same or opposite effects (i.e., activation or repression) on microRNA and target (Figure 1A). This topology determines if a microRNA acts as a buffer for reduction of transcriptional noise [15] or as a so-called ‘expression switch’ (reviewed in [13]). These loops are likely abundant in mammalian GRNs since it has been shown that 44–69% of microRNAs are coordinately regulated with their targets [14]. 

An incoherent FFL (Figure 1B) has been described during development of *Caenorhabditis elegans*, where expression oscillations of the developmental regulator *lin-14* are dampened by pulsatile transcription of the microRNA *lin-4* [16]. *lin-14* is expressed in a temporally graded fashion. However, Kim and colleagues found that its expression becomes periodic in *lin-4* mutants. The periodicity coincides with the pulses of *lin-4* expression. Consequently, it was proposed that the temporal co-expression of *lin-4* and its target *lin-14* leads to the buffering of the expression output. This generates a temporal *lin-14* expression gradient from pulsatile transcription [16].

A developmental switch that determines left-right asymmetry of the two taste receptor neurons in *C. elegans* is also controlled by microRNAs [17,18]. In this case, a double negative feedback loop (Figure 1C) induces the transition from an equipotent precursor state to the fixed bistable expression of specific markers. The two microRNAs *lsy-6* and *miR-273* repress the expression of each other’s transcriptional activators, *die-1* and *cog-1* [19].

The coupling of expression of a target gene and its microRNA could be problematic under circumstances where down-regulation of the target is not desired. It might then be necessary to enable decoupling of the expression of both in order to allow for the derepression of the target gene. This is easily achieved when microRNA and target gene expression are controlled by distinct enhancers that only lead to co-expression under certain circumstances. However, it is more complicated when an intronic or exonic microRNA targets its own host gene (Figure 1D). Bioinformatic analyses in human, mouse, *Drosophila*, and *C. elegans* revealed that indeed 33–52% of microRNAs are located in introns and 0.6–9% in exons of protein-coding genes [20,21] and that 20% of human intragenic microRNAs are predicted to target their own host gene [20]. The transcription of intronic microRNAs is usually thought to be directly linked to that of their host gene [22,23,24,25]. However, 35% of intronic microRNAs have independent regulatory elements [26,27], and their expression can, therefore, differ from that of their host gene. Examples for cases of independent regulation include microRNAs *miR218-1* and *miR218-2* (host genes: *SLIT2* and *SLIT3*) in human and zebrafish [28] and *miR-634* (host gene: *PRKCA*) in human [29]. A well-understood example for a microRNA being co-expressed with its host gene is the *miR-92* family in *Drosophila,* which target their own host gene *jing-interacting gene regulatory 1* (*jigr1*) in order to promote the correct self-renewal of neuroblasts [30]. Expression of *miR-92a* and *miR-92b* is correlated with transcription of *jigr1*. However, alternative mRNA isoforms also allow for expression of *jigr1* alone. Thus, transcription of microRNA and target can be uncoupled when the target gene is expressed as alternative mRNAs which exclude or include the microRNA (Figure 1D). It has indeed been shown that intragenic microRNAs are preferentially located in the 5′ region of their host genes, and that host genes contain more introns than genes without intronic microRNAs [20]. Moreover, the 5′ introns are significantly longer than in a cohort of randomly sampled genes [20]. These findings indicate that alternative transcriptional start sites and regulatory regions could be used in genes with intronic microRNAs to decouple the expression of the microRNA and the host gene.

## 3. microRNA Targets in GRNs

There are many examples where individual microRNAs have been experimentally demonstrated to regulate the transcripts of particular individual genes in a given context. For example, *miR-2* regulation of *Kr-h1* during metamorphosis in hemimetabolous insects [32,33], the requirement of *miR-57* repression of *nob-1* for posterior specification in *C. elegans* [34], and *miR-133* regulation of *Gli3* during vertebrate skeletal myogenesis [35]. 

Some genes—so-called “target hubs”—have been shown to be targeted by several microRNAs [36]. Interestingly, the target hub gene set is enriched for TFs and developmental processes [36]. This suggests that groups of microRNAs target important nodes in GRNs to regulate their outcome. Especially in cases where a gene is expressed in different tissues or under control of a ubiquitous enhancer, targeting by several microRNAs can help to fine-tune this gene’s expression level in different contexts. For example, *p21* encodes a tumour suppressor that is required for cell cycle arrest under different conditions. Expression of *p21* is in vitro down-regulated by 28 different microRNAs [37]. Computational analysis and modelling suggest that indeed, *p21* is repressed by different microRNAs in different contexts to allow progression of the cell cycle [38]. Several of these microRNAs are strongly expressed in different types of cancers [39,40,41,42,43,44], which might, in turn, modulate *p21* levels and thus lead to cancer progression. The expression level of *p21* in different non-pathological contexts could also be regulated by different microRNAs. 

Multiple microRNAs have also been found to act in concert during epithelial to mesenchymal transition (EMT). Cursons and colleagues (2018) demonstrated that microRNAs act in combination with TFs to reinforce transcriptional changes which are required for EMT, and to buffer those changes which are not required [45]. Moreover, the authors showed that multiple microRNAs act in a combinatorial fashion on transcripts. Overexpression of single microRNAs resulted in the non-specific targeting of genes not involved in EMT and, thus, had off-target effects. On the other hand, low-level expression of microRNA combinations was sufficient to induce EMT [45]. These results indicate that synergistically acting microRNAs can reinforce each other and, thus, ensure the required posttranscriptional regulation. Moreover, only a low level of each individual microRNA is necessary which could reduce potential off-target effects of stronger microRNA expression.

As well as multiple microRNAs targeting particular genes in GRNs, individual microRNAs often target multiple genes. Indeed, some microRNAs are predicted to have hundreds of targets, although there are likely to be false positives depending on the stringency of search criteria [1,5,46]. This illustrates the importance of individual microRNAs for certain developmental processes since they can have different targets in various tissues at different stages of development. Moreover, individual microRNAs have been shown to target the transcripts of multiple genes in the same GRN. This may provide robustness to GRNs to ensure precise outputs under different physiological or environmental conditions or in different genetic backgrounds [47,48,49]. 

In vertebrates, one of the roles of *miR-9* is to regulate the transition of progenitor cells from non-neurogenic to neurogenic by promoting differentiation and repressing proliferation (reviewed in [50]). It is thought that *miR-9* does this through regulation of multiple target genes in this GRN including the TFs *Hes1, FoxG1, Gsx2, Zic5* and the nuclear receptor *Tlx/Nr2e1*, which promote proliferation [50,51,52,53,54,55,56]. Moreover, *miR-9* appears to target genes with different functions in this context including factors that help modulate chromatin modifications like repressor-element-1 silencing transcription factor [50,57]. Such targeting at multiple levels might ensure robustness to the overall process.

In *Drosophila*, *miR-9a* also targets different genes in the same gene regulatory pathway to ensure robust control of cell fate. In this case, *miR-9a* is expressed in non-sensory organ precursor cells and helps to specify the correct number of sensory organ precursor (SOP) cells [47,50,58]. Loss of *mir-9a* results in the production of extra sensory neurons [58]. *miR-9a* promotes non-SOP fate through direct repression of pro-neural genes including *senseless* and *Drosophila LIM-only* (*dLMO*) [47,58,59,60]. Interestingly, other members of the *miR-9* family may target other genes during this cell fate decision in *Drosophila* to provide further robustness [50].

**Figure 2:**
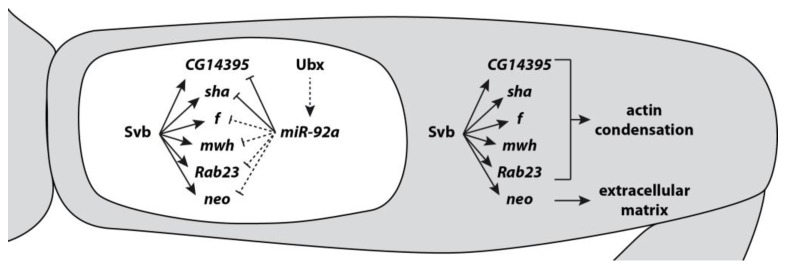
**Trichome formation on *Drosophila* legs is repressed by *miR-92a*.** The TF Shavenbaby (Svb) controls the expression of multiple genes whose products lead to the formation of trichomes (grey) via promotion of actin condensation and changes in the extracellular matrix. Some of these genes (*sha* and *CG14395*) have been shown to be repressed by *miR-92a*, and others are likely targets. The targeting of these genes by *miR-92a* results in a robust suppression of trichome development in the proximal region of the second leg femur where *miR-92a* is expressed (the naked valley; white). *miR-92a* expression is possibly activated by Ubx.

Also in *Drosophila*, the *miR-92* family is involved in the regulation of several developmental processes including circadian rhythm, germline specification, neurogenesis, and trichome patterning, and some of its target genes have been identified [7,30,61,62,63]. In *Drosophila* second legs, *miR-92a* represses trichome formation resulting in a patch of trichome-free cuticle on the proximal region of the femur—the so-called ‘naked valley’ [61,64]. It was shown that *miR-92a* targets the mRNA of *shavenoid* (*sha*) to block trichome formation [61,63]. However, over-expression of *sha* does not produce completely normal trichomes and in addition *CG14395*, another likely direct target gene of *miR-92a*, appears to be required [65]. Intriguingly, several other genes involved in trichome formation that are directly activated by the TF Shavenbaby (Svb) [66,67] are also predicted to be *miR-92a* targets (Franke, Arif, Kittelmann and McGregor unpublished data) using TargetScan [4] (Figure 2). This suggests that in the GRN for leg trichome patterning, *miR-92a* targets multiple genes with different roles in the production of trichomes to ensure robust repression of these structures, thus playing an antagonistic role to Svb. 

Recently it has also been shown that along with *miR-iab4* [68] and *miR-iab8* [69,70], *miR-310C* regulates *Ultrabithorax* (*Ubx*) during haltere development in *Drosophila* [71]. While this indicates that *Ubx* is regulated by multiple microRNAs during the development of this appendage, *miR-310C* has *Ubx*-independent roles in haltere growth and patterning suggesting that it regulates multiple genes in the GRN for haltere development.

## 4. Evolution of microRNAs and Targets Leading to Phenotypic Change

Since the discovery of microRNAs and their role in regulating gene expression, it has been thought that evolutionary changes in microRNA genes have made an important contribution to the diversification of animals [8,9]. These changes may lead to variation in their expression, copy number, arm usage, and seed sequences allowing them to acquire new targets or altering the expression of pre-existing target genes [8]. Indeed, many studies have found an association between the evolution of microRNAs and phenotypic changes among animals including the diversification of cichlids [72] and *Lepidoptera* [73] and even brain function in humans, e.g., [74,75]. However, there are relatively few described cases of phenotypic change in animals where the causal evolutionary changes have been localized to microRNAs.

One exception is again the role of *miR-92a* in trichome patterning in *Drosophila*. The size of the naked valley (see above) varies among different *Drosophila* species and between strains of *D. melanogaster* [61,64]. Genetic mapping combined with analyses of gene expression and function has shown that intra-species variation is caused by changes in the spatial expression of *miR-92a*. Expression of *miR-92a* is proximally restricted and represses *sha* and other trichome genes only in the proximal part of the femur (Figure 2). Further proximal restriction of the expression pattern in some *Drosophila* strains results correspondingly in a smaller naked valley [61]. Although the causative nucleotides have not yet been identified, it is thought that the expression variation has been caused by cis-regulatory changes in *miR-92a* enhancers [61].

Given that changes in the expression of TFs underlie many examples of phenotypic evolution (reviewed in [76,77]), the question arises why are there are so few known examples to date of changes in microRNA genes causing phenotypic diversification among animals? The hardwiring of microRNAs into GRNs allows them to provide robustness and so it may follow that the GRN is likely to be robust to changes in the expression of a given microRNA. In addition, the effect of a microRNA on the expression of individual target genes is often thought to be relatively subtle [78,79], and so it is unlikely that changes in an individual interaction, for example through the evolution of the location or level of expression of the microRNA, will have a detectable phenotypic effect. 

Perhaps then it is only in specific developmental contexts with GRNs of particular topography where a microRNA targets the mRNAs of multiple genes required for a given developmental outcome that changes in the expression of the microRNA could result in phenotypic evolution [65]. As Bartel (2018) has surmised, our understanding of the functions of many microRNAs is usually based on experimental evidence of their effect on one or a few target genes [1]. Therefore, it is probable that, as in the cases of *miR-92a* in trichome development and *miR-9a* in SOP specification, more examples will emerge of microRNAs targeting multiple genes in the same GRNs. Such a better understanding of microRNA targets in GRNs combined with higher resolution genetic mapping of phenotypic changes could reveal many more examples of changes in microRNAs causing developmental and phenotypic evolution.

## References

[1] Bartel D.P. (2018). Metazoan MicroRNAs. Cell.

[2] Mukherji S., Ebert M.S., Zheng G.X., Tsang J.S., Sharp P.A., van Oudenaarden A. (2011). MicroRNAs can generate thresholds in target gene expression. Nat. Genet..

[3] Posadas D.M., Carthew R.W. (2014). MicroRNAs and their roles in developmental canalization. Curr. Opin. Genet. Dev..

[4] Agarwal V., Subtelny A.O., Thiru P., Ulitsky I., Bartel D.P. (2018). Predicting microRNA targeting efficacy in Drosophila. Genome Biol..

[5] Friedman R.C., Farh K.K., Burge C.B., Bartel D.P. (2009). Most mammalian mRNAs are conserved targets of microRNAs. Genome Res..

[6] Jan C.H., Friedman R.C., Ruby J.G., Bartel D.P. (2011). Formation, regulation and evolution of Caenorhabditis elegans 3’UTRs. Nature.

[7] Chen Y.W., Song S., Weng R., Verma P., Kugler J.M., Buescher M., Rouam S., Cohen S.M. (2014). Systematic study of Drosophila microRNA functions using a collection of targeted knockout mutations. Dev. Cell.

[8] Berezikov E. (2011). Evolution of microRNA diversity and regulation in animals. Nat. Rev. Genet..

[9] Niwa R., Slack F.J. (2007). The evolution of animal microRNA function. Curr. Opin. Genet. Dev..

[10] Alberti C., Cochella L. (2017). A framework for understanding the roles of miRNAs in animal development. Development.

[11] Liu N., Okamura K., Tyler D.M., Phillips M.D., Chung W.J., Lai E.C. (2008). The evolution and functional diversification of animal microRNA genes. Cell Res..

[12] Bartel D.P., Chen C.Z. (2004). Micromanagers of gene expression: The potentially widespread influence of metazoan microRNAs. Nat. Rev. Genet..

[13] Cora D., Re A., Caselle M., Bussolino F. (2017). MicroRNA-mediated regulatory circuits: Outlook and perspectives. Phys. Biol..

[14] Tsang J., Zhu J., van Oudenaarden A. (2007). MicroRNA-mediated feedback and feedforward loops are recurrent network motifs in mammals. Mol. Cell.

[15] Osella M., Bosia C., Cora D., Caselle M. (2011). The role of incoherent microRNA-mediated feedforward loops in noise buffering. PLoS Comput. Biol..

[16] Kim D., Grun D., van Oudenaarden A. (2013). Dampening of expression oscillations by synchronous regulation of a microRNA and its target. Nat. Genet..

[17] Chang S., Johnston R.J., Frokjaer-Jensen C., Lockery S., Hobert O. (2004). MicroRNAs act sequentially and asymmetrically to control chemosensory laterality in the nematode. Nature.

[18] Johnston R.J., Hobert O. (2003). A microRNA controlling left/right neuronal asymmetry in Caenorhabditis elegans. Nature.

[19] Johnston R.J., Chang S., Etchberger J.F., Ortiz C.O., Hobert O. (2005). MicroRNAs acting in a double-negative feedback loop to control a neuronal cell fate decision. Proc. Natl. Acad. Sci. USA.

[20] Hinske L.C., Galante P.A., Kuo W.P., Ohno-Machado L. (2010). A potential role for intragenic miRNAs on their hosts’ interactome. BMC Genom..

[21] Hinske L.C., Franca G.S., Torres H.A., Ohara D.T., Lopes-Ramos C.M., Heyn J., Reis L.F., Ohno-Machado L., Kreth S., Galante P.A. (2014). miRIAD-integrating microRNA inter- and intragenic data. Database J. Biol. Databases Curation.

[22] Baskerville S., Bartel D.P. (2005). Microarray profiling of microRNAs reveals frequent coexpression with neighboring miRNAs and host genes. RNA.

[23] Rodriguez A., Griffiths-Jones S., Ashurst J.L., Bradley A. (2004). Identification of mammalian microRNA host genes and transcription units. Genome Res..

[24] Gennarino V.A., Sardiello M., Avellino R., Meola N., Maselli V., Anand S., Cutillo L., Ballabio A., Banfi S. (2009). MicroRNA target prediction by expression analysis of host genes. Genome Res..

[25] Liang Y., Ridzon D., Wong L., Chen C. (2007). Characterization of microRNA expression profiles in normal human tissues. BMC Genom..

[26] Ozsolak F., Poling L.L., Wang Z., Liu H., Liu X.S., Roeder R.G., Zhang X., Song J.S., Fisher D.E. (2008). Chromatin structure analyses identify miRNA promoters. Genes Dev..

[27] Monteys A.M., Spengler R.M., Wan J., Tecedor L., Lennox K.A., Xing Y., Davidson B.L. (2010). Structure and activity of putative intronic miRNA promoters. RNA.

[28] Punnamoottil B., Rinkwitz S., Giacomotto J., Svahn A.J., Becker T.S. (2015). Motor neuron-expressed microRNAs 218 and their enhancers are nested within introns of Slit2/3 genes. Genesis.

[29] Paraboschi E.M., Cardamone G., Rimoldi V., Duga S., Solda G., Asselta R. (2017). miR-634 is a Pol III-dependent intronic microRNA regulating alternative-polyadenylated isoforms of its host gene PRKCA. Biochim. Biophys. Acta Gen. Subj..

[30] Yuva-Aydemir Y., Xu X.L., Aydemir O., Gascon E., Sayin S., Zhou W., Hong Y., Gao F.B. (2015). Downregulation of the Host Gene jigr1 by miR-92 Is Essential for Neuroblast Self-Renewal in Drosophila. PLoS Genet..

[31] Herranz H., Cohen S.M. (2010). MicroRNAs and gene regulatory networks: Managing the impact of noise in biological systems. Genes Dev..

[32] Belles X. (2017). MicroRNAs and the Evolution of Insect Metamorphosis. Annu. Rev. Entomol..

[33] Lozano J., Montanez R., Belles X. (2015). MiR-2 family regulates insect metamorphosis by controlling the juvenile hormone signaling pathway. Proc. Natl. Acad. Sci. USA.

[34] Zhao Z., Boyle T.J., Liu Z., Murray J.I., Wood W.B., Waterston R.H. (2010). A negative regulatory loop between microRNA and Hox gene controls posterior identities in Caenorhabditis elegans. PLoS Genet..

[35] Mok G.F., Lozano-Velasco E., Maniou E., Viaut C., Moxon S., Wheeler G., Munsterberg A. (2018). miR-133-mediated regulation of the Hedgehog pathway orchestrates embryo myogenesis. Development.

[36] Shalgi R., Lieber D., Oren M., Pilpel Y. (2007). Global and local architecture of the mammalian microRNA-transcription factor regulatory network. PLoS Comput. Biol..

[37] Wu S., Huang S., Ding J., Zhao Y., Liang L., Liu T., Zhan R., He X. (2010). Multiple microRNAs modulate p21Cip1/Waf1 expression by directly targeting its 3’ untranslated region. Oncogene.

[38] Lai X., Schmitz U., Gupta S.K., Bhattacharya A., Kunz M., Wolkenhauer O., Vera J. (2012). Computational analysis of target hub gene repression regulated by multiple and cooperative miRNAs. Nucleic Acids Res..

[39] Wong T.S., Liu X.B., Wong B.Y., Ng R.W., Yuen A.P., Wei W.I. (2008). Mature miR-184 as Potential Oncogenic microRNA of Squamous Cell Carcinoma of Tongue. Clin. Cancer Res. Off. J. Am. Assoc. Cancer Res..

[40] Jongen-Lavrencic M., Sun S.M., Dijkstra M.K., Valk P.J., Lowenberg B. (2008). MicroRNA expression profiling in relation to the genetic heterogeneity of acute myeloid leukemia. Blood.

[41] Lionetti M., Biasiolo M., Agnelli L., Todoerti K., Mosca L., Fabris S., Sales G., Deliliers G.L., Bicciato S., Lombardi L. (2009). Identification of microRNA expression patterns and definition of a microRNA/mRNA regulatory network in distinct molecular groups of multiple myeloma. Blood.

[42] Voorhoeve P.M., le Sage C., Schrier M., Gillis A.J., Stoop H., Nagel R., Liu Y.P., van Duijse J., Drost J., Griekspoor A. (2006). A genetic screen implicates miRNA-372 and miRNA-373 as oncogenes in testicular germ cell tumors. Cell.

[43] Guled M., Lahti L., Lindholm P.M., Salmenkivi K., Bagwan I., Nicholson A.G., Knuutila S. (2009). CDKN2A, NF2, and JUN are dysregulated among other genes by miRNAs in malignant mesothelioma-A miRNA microarray analysis. Genes Chromosom. Cancer.

[44] Gottardo F., Liu C.G., Ferracin M., Calin G.A., Fassan M., Bassi P., Sevignani C., Byrne D., Negrini M., Pagano F. (2007). Micro-RNA profiling in kidney and bladder cancers. Urol. Oncol..

[45] Cursons J., Pillman K.A., Scheer K.G., Gregory P.A., Foroutan M., Hediyeh-Zadeh S., Toubia J., Crampin E.J., Goodall G.J., Bracken C.P. (2018). Combinatorial Targeting by MicroRNAs Co-ordinates Post-transcriptional Control of EMT. Cell Syst..

[46] Pinzon N., Li B., Martinez L., Sergeeva A., Presumey J., Apparailly F., Seitz H. (2017). microRNA target prediction programs predict many false positives. Genome Res..

[47] Cassidy J.J., Jha A.R., Posadas D.M., Giri R., Venken K.J., Ji J., Jiang H., Bellen H.J., White K.P., Carthew R.W. (2013). miR-9a minimizes the phenotypic impact of genomic diversity by buffering a transcription factor. Cell.

[48] Cassidy J.J., Straughan A.J., Carthew R.W. (2016). Differential Masking of Natural Genetic Variation by miR-9a in Drosophila. Genetics.

[49] Li X., Cassidy J.J., Reinke C.A., Fischboeck S., Carthew R.W. (2009). A microRNA imparts robustness against environmental fluctuation during development. Cell.

[50] Coolen M., Katz S., Bally-Cuif L. (2013). miR-9: A versatile regulator of neurogenesis. Front. Cell. Neurosci..

[51] Bonev B., Pisco A., Papalopulu N. (2011). MicroRNA-9 reveals regional diversity of neural progenitors along the anterior-posterior axis. Dev. Cell.

[52] Coolen M., Thieffry D., Drivenes O., Becker T.S., Bally-Cuif L. (2012). miR-9 controls the timing of neurogenesis through the direct inhibition of antagonistic factors. Dev. Cell.

[53] Bonev B., Stanley P., Papalopulu N. (2012). MicroRNA-9 Modulates Hes1 ultradian oscillations by forming a double-negative feedback loop. Cell Rep..

[54] Tan S.L., Ohtsuka T., Gonzalez A., Kageyama R. (2012). MicroRNA9 regulates neural stem cell differentiation by controlling Hes1 expression dynamics in the developing brain. Genes Cells Devot. Mol. Cell. Mech..

[55] Shibata M., Kurokawa D., Nakao H., Ohmura T., Aizawa S. (2008). MicroRNA-9 modulates Cajal-Retzius cell differentiation by suppressing Foxg1 expression in mouse medial pallium. J. Neurosci. Off. J. Soc. Neurosci..

[56] Zhao C., Sun G., Li S., Shi Y. (2009). A feedback regulatory loop involving microRNA-9 and nuclear receptor TLX in neural stem cell fate determination. Nat. Struct. Mol. Biol..

[57] Packer A.N., Xing Y., Harper S.Q., Jones L., Davidson B.L. (2008). The bifunctional microRNA miR-9/miR-9* regulates REST and CoREST and is downregulated in Huntington’s disease. J. Neurosci. Off. J. Soc. Neurosci..

[58] Li Y., Wang F., Lee J.A., Gao F.B. (2006). MicroRNA-9a ensures the precise specification of sensory organ precursors in Drosophila. Genes Dev..

[59] Biryukova I., Asmar J., Abdesselem H., Heitzler P. (2009). Drosophila mir-9a regulates wing development via fine-tuning expression of the LIM only factor, dLMO. Dev. Biol..

[60] Bejarano F., Smibert P., Lai E.C. (2010). miR-9a prevents apoptosis during wing development by repressing Drosophila LIM-only. Dev. Biol..

[61] Arif S., Murat S., Almudi I., Nunes M.D., Bortolamiol-Becet D., McGregor N.S., Currie J.M., Hughes H., Ronshaugen M., Sucena E. (2013). Evolution of mir-92a Underlies Natural Morphological Variation in Drosophila melanogaster. Curr. Biol..

[62] Chen X., Rosbash M. (2017). MicroRNA-92a is a circadian modulator of neuronal excitability in Drosophila. Nat. Commun..

[63] Schertel C., Rutishauser T., Forstemann K., Basler K. (2012). Functional Characterization of Drosophila microRNAs by a Novel in vivo Library. Genetics.

[64] Stern D.L. (1998). A role of Ultrabithorax in morphological differences between Drosophila species. Nature.

[65] Kittelmann S., Buffry A.D., Franke F.A., Almudi I., Yoth M., Sabaris G., Couso J.P., Nunes M.D.S., Frankel N., Gomez-Skarmeta J.L. (2018). Gene regulatory network architecture in different developmental contexts influences the genetic basis of morphological evolution. PLoS Genet..

[66] Chanut-Delalande H., Fernandes I., Roch F., Payre F., Plaza S. (2006). Shavenbaby couples patterning to epidermal cell shape control. PLoS Biol..

[67] Menoret D., Santolini M., Fernandes I., Spokony R., Zanet J., Gonzalez I., Latapie Y., Ferrer P., Rouault H., White K.P. (2013). Genome-wide analyses of Shavenbaby target genes reveals distinct features of enhancer organization. Genome Biol..

[68] Ronshaugen M., Biemar F., Piel J., Levine M., Lai E.C. (2005). The Drosophila microRNA iab-4 causes a dominant homeotic transformation of halteres to wings. Genes Dev..

[69] Tyler D.M., Okamura K., Chung W.J., Hagen J.W., Berezikov E., Hannon G.J., Lai E.C. (2008). Functionally distinct regulatory RNAs generated by bidirectional transcription and processing of microRNA loci. Genes Dev..

[70] Stark A., Bushati N., Jan C.H., Kheradpour P., Hodges E., Brennecke J., Bartel D.P., Cohen S.M., Kellis M. (2008). A single Hox locus in Drosophila produces functional microRNAs from opposite DNA strands. Genes Dev..

[71] Kaschula R., Pinho S., Alonso C.R. (2018). MicroRNA-dependent regulation of Hox gene expression sculpts fine-grain morphological patterns in a Drosophila appendage. Development.

[72] Franchini P., Xiong P., Fruciano C., Meyer A. (2016). The Role of microRNAs in the Repeated Parallel Diversification of Lineages of Midas Cichlid Fish from Nicaragua. Genome Biol. Evolut..

[73] Quah S., Hui J.H., Holland P.W. (2015). A Burst of miRNA Innovation in the Early Evolution of Butterflies and Moths. Mol. Biol. Evolut..

[74] Li J., Zhang Z. (2013). miRNA regulatory variation in human evolution. Trends Genet..

[75] Somel M., Liu X., Tang L., Yan Z., Hu H., Guo S., Jiang X., Zhang X., Xu G., Xie G. (2011). MicroRNA-driven developmental remodeling in the brain distinguishes humans from other primates. PLoS Biol..

[76] Martin A., Orgogozo V. (2013). The Loci of repeated evolution: A catalog of genetic hotspots of phenotypic variation. Evolution.

[77] Carroll S.B. (2008). Evo-devo and an expanding evolutionary synthesis: A genetic theory of morphological evolution. Cell.

[78] Selbach M., Schwanhausser B., Thierfelder N., Fang Z., Khanin R., Rajewsky N. (2008). Widespread changes in protein synthesis induced by microRNAs. Nature.

[79] Baek D., Villen J., Shin C., Camargo F.D., Gygi S.P., Bartel D.P. (2008). The impact of microRNAs on protein output. Nature.

